# SARS-CoV-2 disease severity and diabetes: why the connection and what is to be done?

**DOI:** 10.1186/s12979-020-00192-y

**Published:** 2020-06-30

**Authors:** Caio Henrique Mazucanti, Josephine Mary Egan

**Affiliations:** National Institute on Aging, Intramural Research Program, 251 Bayview Boulevard, Baltimore, MD 21224 USA

**Keywords:** COVID-19, Diabetes mellitus (DM), SARS-CoV-2, ACE2

## Abstract

Severe acute respiratory syndrome coronavirus 2 (SARS-CoV-2), a novel virus responsible for the current coronavirus disease 2019 (COVID-19) pandemic, has infected over 3.5 million people all over the world since the first case was reported from Wuhan, China 5 months ago. As more epidemiological data regarding COVID-19 patients is acquired, factors that increase the severity of the infection are being identified and reported. One of the most consistent co-morbidities associated with worse outcome in COVID-19 patients is diabetes, along with age and cardiovascular disease. Studies on the association of diabetes with other acute respiratory infections, namely SARS, MERS, and Influenza, outline what seems to be an underlying factor in diabetic patients that makes them more susceptible to complications. In this review we summarize what we think may be the factors driving this pattern between diabetes, aging and poor outcomes in respiratory infections. We also review therapeutic considerations and strategies for treatment of COVID-19 in diabetic patients, and how the additional challenge of this co-morbidity requires attention to glucose homeostasis so as to achieve the best outcomes possible for patients.

## Introduction

Since the first reported case on December 9, 2019, in Wuhan, China, cases of the coronavirus 2019 disease (COVID-19) quickly escalated to global pandemic levels within a month. At present, 5 months later, the number of confirmed cases surpasses 3.5 million, reported across 200 countries and regions all over the world [1, 2]. The virus responsible for COVID-19, now named SARS-CoV-2, had not previously been described in humans or animals and was reported in January 2020 after isolation from bronchoalveolar fluid of patients [3, 4]. It is a new beta-coronavirus that shares a high percentage of genomic similarities with SARS-CoV, the coronavirus responsible for the severe acute respiratory syndrome (SARS) outbreak in 2003 [4]. Among the respiratory syndromes caused by beta-coronaviruses in humans, SARS-CoV-2 infection has a higher basic reproduction number compared to SARS- CoV and the Middle East respiratory syndrome (MERS)-CoV, representing a greater threat for international public health [5]. Preliminary estimates of mortality rates indicate that SARS-CoV-2 infection is less often fatal than MERS- and SARS-CoV respiratory infections (3.4% against 34.4 and 15%, respectively) [2, 6, 7]. However, as evidenced by its higher reproduction rate, the COVID-19 outbreak already has caused a higher total number of deaths than the other coronavirus respiratory syndromes. While cases of COVID-19 were estimated at around 3.5 million around the world as of May 4th, 2020, with over 250,000 deaths, the 2003 SARS outbreak had only 8000 cases across 29 countries, with 774 total deaths, while the 2012 MERS outbreak had 2494 confirmed cases, reported by 27 countries, with a total of 858 deaths [2, 6, 7]. As an ongoing challenge and with the number of confirmed cases increasing exponentially, epidemiological data characterizing COVID-19 patients are valuable in helping to control the spread and treatment of the disease. While it’s considered a respiratory virus, it is now evident that is invades many organs beside the upper and lower respiratory tract and can cause multisystem-wide disease with characteristics unique to each organ it invades.

To date, studies describing comorbidities related to COVID-19 report correlations with distinctive underlying diseases. In one of the first reports describing the clinical features of hospitalized COVID-19 patients published in February 2020, Huang et al. found that 32% (13 patients) had other health issues, most common being diabetes, cardiovascular disease, hypertension, and chronic obstructive pulmonary disease [8]. The high incidence of diabetes throughout the world makes this truly worrisome as the pandemic has spread. Subsequent studies and broader data collection confirmed such observations. In a study including 1099 patients, 15.7% of whom were manifesting severe symptoms, the most common co-morbidities were hypertension (23.7%), diabetes (16.2%), heart disease (5.8%), and cerebrovascular disease (2.3%) [9]. Hypertension and diabetes were also the most common co-morbidities in a third study that included 140 patients, with a prevalence of 30 and 12%, respectively, where they also correlated with the severity and clinical outcome of the disease [10]. In a study of 138 patients, Wang et al. found that 46.4% (64 patients) had other underlying conditions and, notably, that number is much higher (72.2%) amongst patients in the intensive care unit [11]. In addition, among cases with a negative outcome, Yang et al. described that, of a group of 32 non-survivors, 7 (22%) had cerebrovascular diseases, and 7 had diabetes [12]. In a systematic review including eight different studies, encompassing a total of 46,248 confirmed cases, Yang et al. found that the most common co-morbidities among COVID-19 patients were hypertension (17%) and diabetes (8%). Patients with other underlying respiratory diseases amounted to only 2% [13]. A summary report from the Chinese Center for Disease Control of 72,314 cases, the largest case load to date, informs us that the overall fatality from SARS-CoV-2 is 2.3% but that percentage is 7.3% in those with diabetes [14]. So, in sum, while evidence that diabetes per se makes it more likely that one gets infected with SARS-CoV-2 is inconclusive or lacking altogether (because we lack information on the group of people who get infected but have no symptoms), the diabetic condition makes it more likely that infection will be more severe and therefore will result in more hospitalizations, that hospitalized diabetic patients will need more intensive care, that diabetes patients will spend a longer time in hospital until they are discharged, and that patients with diabetes are more likely to die than are non-diabetic patients.

## Pathophysiology of infections in people with diabetes

### Generalized immunological findings with viral infections in diabetic patients

**Fig. 1:**
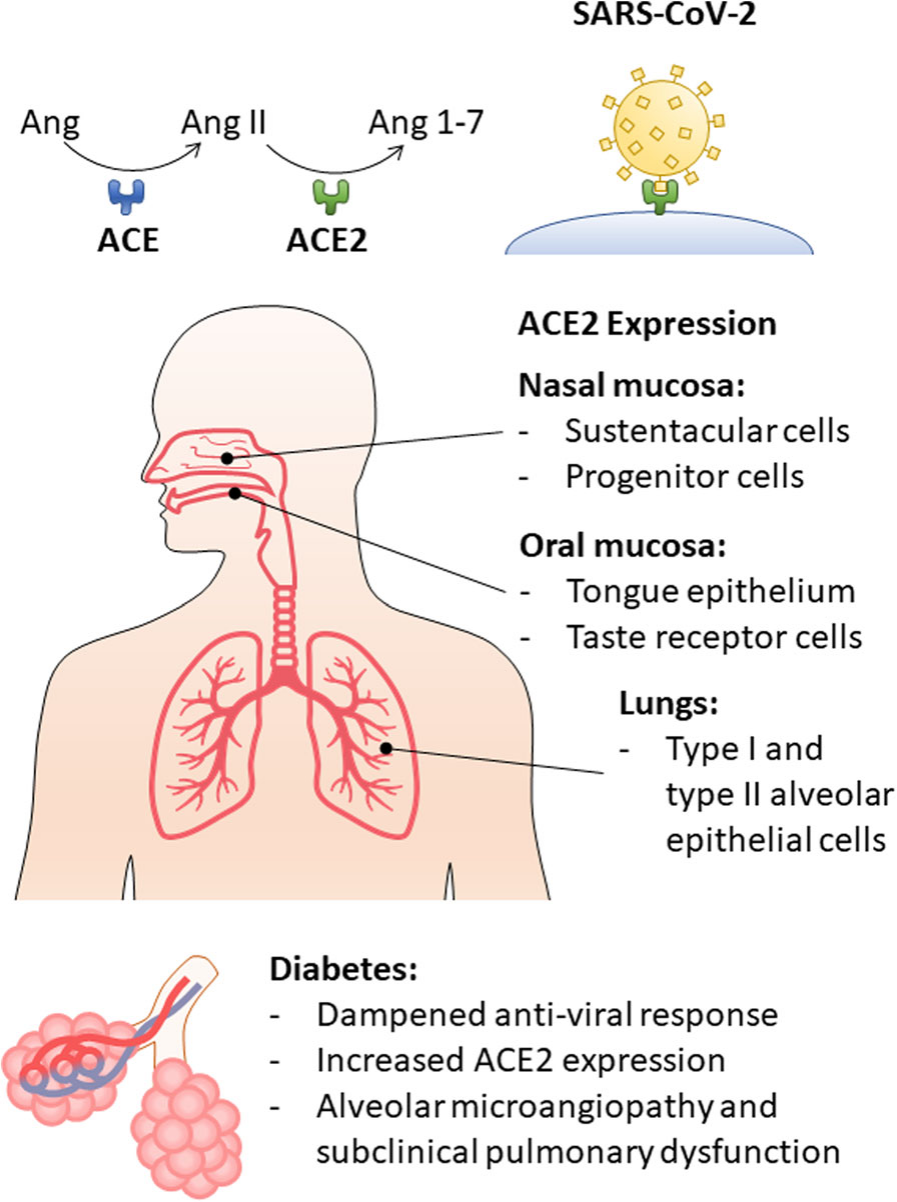
SARS-CoV-2 sites of infection and complications in diabetic patients. As is the case for SARS-CoV, SARS-CoV-2 uses the ectopeptidase ACE2 as an entry site in human host cells. ACE2 is part of the renin-angiotensin system, responsible for the termination of the angiotensin signal, promoting conversion of angiotensin II (Ang II) into angiotensin 1–7 (Ang 1–7). Ang II acts through the angiotensin receptor 1 (AT1), which results in vasoconstriction and sympathetic nervous stimulation. Ang 1–7, on the other hand, acts on angiotensin receptor 2 (AT2), and has opposite effects, inducing vasodilation. ACE2 is expressed throughout the respiratory epithelium, from the nasal and oral mucosa to the epithelium in alveoli. Diabetic patients are at higher risk of infection and complications from COVID-19 as a result of several factors. Dampened anti-viral response with reduced interferon production is a common feature seen in persons with diabetes. Additionally, diabetic patients may be more vulnerable to SARS-CoV-2 infection due to elevated ACE2 expression. Finally, diabetes-related subclinical pulmonary dysfunction and microvascular disease may be aggravating factors that contribute to the severity of the respiratory symptoms associated with COVID-19

It is known that diabetic patients, especially with uncontrolled glycemia, are at higher risk to contract infections, a trend that correlates tightly with glycated hemoglobin levels. In clinical practice, higher incidence of foot infections, yeast infections, urinary tract infections, and surgical site infections is commonly seen in diabetic patients [15–18]. There are several host factors that can explain this observation, ranging from local changes in bacterial colonization, to systemic alterations in immune response. For instance, cytokine release by macrophages and T-cells is disturbed in diabetes, which also impairs neutrophil recruitment [19]. Alteration of innate immune responses and humoral innate immunity were observed both in vitro and in diabetic patients [20–22]. In non-obese diabetic mice, basal levels of interferon-α (IFN-α), a type I interferon, are reduced when compared to pre-diabetic mice, and IFN-γ production by CD8+ T cells is impaired [21]. Similarly, in persons with either type 1 or type 2 diabetes (T1DM and T2DM, respectively), IFN-α production by dendritic cells is reduced [22]. Impaired dendritic cell functions, together with reduced activation of natural killer cells, compromise proper adaptative immune responses (Fig. 1).

In the adipose tissue of T2DM mouse models and human patients, regulatory or anti-inflammatory macrophages (M2) and regulatory T cells (Treg) change their profile to pro-inflammatory macrophages (M1) and Th1 and Th17 CD4+ T cells [23]. As such, delayed activation of Th1 response and a late hyper-inflammatory reaction are regular findings in diabetes [24]. These alternations in immune profile likely contribute to the reasons why patients with DM have higher incidence and severity of infections.

Type I IFN plays a crucial role in CD4+ T cells differentiation and antiviral response [25]. Dendritic cells are a necessary element to induce type I IFN-dependent polarization of CD4+ T cells into follicular helper T cells (Tfh), vital for immune humoral responses against viral infections [26]. Interestingly, CD4+ T cell polarization into Tfh happens with IFN exposure at early stages of the infection, while exposure at delayed stages promotes differentiation into Th1 cells [26]. As mentioned above, reduced levels of IFN-α production by dendritic cells is a feature found in both T1DM and T2DM patients, which may favor Th1 differentiation to the detriment of Tfh.

While the SARS-CoV responsible for the 2003 outbreak is able to suppress the induction of type I IFN in dendritic cells by inhibiting the transcription factor IRF-3 (interferon regulatory transcription factor-3) [27], this does not seem to be the case for SARS-CoV-2 [28]. In fact, Lokugamage and colleagues show that SARS-CoV-2 is much more sensitive to type I IFN priming than SARS-CoV, as measured by reduced viral protein and replication in Vero E6 cells [28]. As such, the reduced basal levels of type I IFN observed in diabetic patients offer an additional explanation for the increased risk and severity of COVID-19. Human IFNα2b by vapor inhalation is a adjuvant treatment recommended in guidelines for treatment of coronaviruses in China [29]. At least two on-going clinical trials (NCT04276688 and ChiCTR2000029387) are now evaluating two different type I IFNs (IFNβ1b and IFNα2b, respectively) in combination of other antiviral drugs for the treatment of COVID-19. In an investigator-initiated open-label study, human recombinant IFNα nasal drops seemed to effectively prevent infection in medical personnel who were likely to be exposed to SARS-CoV-2, indicating that IFNα inhalation has promise for protecting susceptible healthy people during the coronavirus pandemic, and underlining the importance of IFN in this disease [30]. If this report is replicated, it would be a hugely significant advance in fighting this disease.

### Diabetes, aging, and immune function

According to the 2020 National Diabetes Statistics Report, elaborated and published by the CDC, 13% of all US adults (age 18 and up) have diabetes, corresponding to 10.4% of total population. Additionally, the prevalence of diabetes increases with age. While 4.3% of adults between 18 and 44 years of age have diabetes, this number increases to 17.5% in the age range of 45–64 years, and to 26.8% in people 65 and above [31]. Even though elderly individuals with DM get infections that are similar to common infections found in younger DM patients, age-related immune senescence is an additional component to the already compromised cell-mediated immunity seen in diabetic persons.

Several different aspects of the immune response are altered during aging. Of those, changes in T-cell mediated response are the most studied and best described. Due to gradual thymic involution with aging, production of naive T cells is decreased, resulting in a limited diversity of T-cell receptors apt to recognize new antigens. As such, even though the total number of T cells is mostly unaltered with age, there is a higher prevalence of memory T lymphocytes in older individuals, to the detriment of naive cells [32]. This characteristic is one of the reasons why elderly persons have an adequate immune response to previously exposed antigens but poorer response when exposed to new ones.

Decreased number of naive T cells, however, is only one aspect of the T-cell system that is altered with aging. Expression of IL2 and its receptor, IL2R, and signal transduction are decreased with aging [33], and a number of other steps in T-cell maturation and activation are also affected One factor that is particularly interesting in the context of diabetes is the association of type I IFNs and T-cell differentiation and survival.

As previously mentioned, type I IFNs produced by dendritic cells are a crucial element in CD4+ T cells differentiation and antiviral response. When dendritic cells present antigen molecules to antigen-specific T cells, there is clonal expansion of these lymphocytes and subsequent differentiation into effector cells, some of which will, later, establish T-cell memory. In elderly individuals this process is compromised.

Li and colleagues [34] found that, even though the expression of IFN receptor and elements of its intracellular signaling (JAK-STAT pathway) did not change with age, type I IFN response was still diminished in activated naive CD4+ T cells from older individuals. Rather than decreased expression of components of the IFN response, they found an upregulation of inhibitory elements. Thus, CD4+ T cells of elderly persons can be considered resistant to TNF, contributing to impaired cell-mediate immune response. This may represent an additional burden in elderly individuals with DM since, as described above, IFN-α production by dendritic cells is reduced in T2DM, which may have therapeutic implications.

### Diabetes and respiratory infections

Aside from bacterial and yeast infections, a correlation between diabetes and the prevalence and severity of viral respiratory infections are also well described. People with either T1DM or T2DM are reported to have a higher risk of serious complications from the common flu [35], caused by different influenza viruses, members of the myxovirus family. According to the Centers for Disease Control and Prevention (CDC), around 30% of patients hospitalized due to common flu-related complications had diabetes. The 2009 swine flu pandemic, caused by an outbreak of influenza A virus subtype H1N1 (A/H1N1), showed similar numbers. A Canadian study with 239 hospitalized patients with PCR-confirmed influenza A/H1N1 found that diabetes triples the risk of hospitalization and, once hospitalized, diabetic patients had quadruple the risk of being admitted to intensive care [36].

Diabetes and plasma glucose levels were also correlated with severity and mortality in SARS patients [37]. In a retrospective analysis, Yang et al. compared data from over 400 patients, and found that fasting plasma glucose levels were higher in SARS-positive patients than in patients with no-SARS pneumonia. Interestingly, in patients who had died from SARS infection, plasma glucose levels were even higher. Similarly, 21.5% of the patients who had a negative outcome from SARS infection had a history of diabetes, a percentage significantly higher than those who recovered from the infection (3.9%).

Of particular interest right now, Kulcsar and colleagues studied the severity of MERS-CoV infection in a mouse model of diabetes induced by a high-fat diet [38]. Their results show a more severe and prolonged acute phase of infection in diabetic mice, with impaired CD4+ T cell recruitment to the lungs and altered cytokine profiles, such as elevated IL17. A similar inflammatory response is observed in COVID-19 patients as well, where low CD4+ T cell counts are seen together with a disproportionally high number of Th17 cells. As mentioned previously, one characteristic of the cell-mediate response during aging is decreased IL2 production and signaling. This also favors CD4+ T cell differentiation into Th17 cells [39]. In addition, this inflammatory profile is accentuated in diabetes, and may go a long way towards explaining the severity of COVID-19 in diabetic patients [40].

### The diabetic lung

An often-neglected characteristic of diabetes is its effects on the lungs. One common finding in diabetic patients is abnormalities in small vessels, with thickening of capillary basement membrane that affects many organs and tissues. This condition, known as diabetic microangiopathy or microvascular disease, is an important factor to determine the prognosis of diabetic patients, and it frequently affects kidneys, eyes, skin, and muscle. Another important target, however, and particularly relevant to this discussion, is the lungs. Alveolar diabetic microangiopathy usually presents itself as subclinical pulmonary dysfunction under normal conditions (Fig. 1), since alveolar microvasculature is structured with large physiological reserve. However, even in otherwise healthy diabetic patients, restriction of lung volume and diffusing capacity can be found in both T1DM and T2DM [41]. In a cross-sectional study of 69 patients, peak oxygen uptake, pulmonary blood flow, diffusing capacity, and capillary blood volume were all found to be significantly reduced in nonsmoking T2DM patients during exercise. This functional impairment correlated directly with glycemic control (as measured by HbA1C levels), extrapulmonary microangiopathy (retinopathy, neuropathy, and microalbuminuria), and was aggravated by obesity [41].

In a study with obese diabetic animals, Foster and colleagues found extensive fat deposits in the lungs, septal lipofibroblasts, interstitial matrix, and the alveolar macrophages, which correlated with alterations in the interstitial environment that presented as increased number of interstitial cells, matrix and collagen, and reduced capillary volume with thickening of capillary basement membrane [42]. Intracellular lipid droplets in macrophages is a hallmark feature of classical M1 activation, which is caused by a profound cellular metabolic shift towards aerobic glycolysis and decreased mitochondrial respiration [43]. Diabetes, in this case, stimulates macrophage activation in response to sterile inflammation, which may have profound consequences in the proper inflammatory response to infections, as discussed above.

Another point of concern in diabetic patients regarding their pulmonary function is broncho-motor tone and control of ventilation, since autonomic neuropathy is a common finding that can be present in up to 30% of patients [44]. Impaired noradrenergic innervation in bronchioles occurs in diabetic patients with autonomic neuropathy despite apparent normality in respiratory functions [45]. Loss of autonomic innervation, however, may alter respiratory response to endogenous and exogenous stimuli. As such, it is very plausible that this damage has a negative impact on both the course and prognosis of respiratory infections.

### Diabetes and SARS-CoV-2 infection

Even though a disturbance in immune response may explain the higher risk of infection and worse outcome of diabetic patients with influenza, the relationship between diabetes and respiratory infections caused by the coronaviruses MERS-CoV, SARS-CoV and SARS-CoV-2 may be more complicated. The first step in the process of viral infection is the attachment of the virus to its targeted cells. There are seven known human coronaviruses, all capable of infecting cells in the respiratory system: HCoV-OC43 and HCoV-229E were first described in the 1960s, and are thought to cause the common cold [46]; SARS-CoV, identified in 2003 [47]; HCoV-NL63 and HCoV-HKU1 in 2004, associated with weak respiratory infections [48]; MERS-CoV, described in 2012 [49]; and now SARS-CoV-2, responsible for the ongoing COVID-19 pandemic.

In these coronaviruses, the cellular attachment and targeting is mediated by a glycoprotein, called spike glycoprotein, found in the enveloped surface of the virus. The interaction of this glycoprotein with specific molecular targets found in host cells allows for viral adhesion and, ultimately, cellular infection. Some of these targets were identified for human coronaviruses and, interestingly, all of them are membrane-bound exopeptidases. The aminopeptidase N (APN), a metalloprotease, mediates the attachment of HCoV-229E to host cells [50]. The angiotensin-converting enzyme 2 (ACE2), responsible for the hydrolysis of angiotensin II into angiotensin 1–7, is the co-receptor for SARS-CoV and HCoV-NL63 [51, 52]. Finally, dipeptidyl peptidase 4 (DPP-4), a transmembrane glycoprotein, allows the attachment of MERS-CoV to human host cells, but not SARS-CoV-2 [53, 54].

Different studies now suggest that, besides SARS-CoV and HCoV-NL63, ACE2 is also the host co-receptor for SARS-CoV-2 (Fig. 1). In in vitro studies, using HeLa cells with and without human ACE2 expression, Zhou et al. showed that the novel 2019 coronavirus can only infect those cells that express ACE2 [55]. Furthermore, Wrapp et al. reported the binding kinetics of SARS-CoV-2 to human ACE2, showing a strong and specific binding with an affinity 20x higher when compared to SARS-CoV [56]. As such, the association between ACE2 expression and SARS-CoV-2 infection vulnerability seems clear.

In two different mouse models of diabetes, ACE2 expression levels are increased in different organs, including the lungs [57, 58]. Recently, a similar finding was described in humans a well [54]. In a phenome-wide Mendelian randomization study investigating factors that causally correlate with pulmonary ACE2 expression, Rao et al. found that increased ACE2 expression had the most consistent causal link with T2DM traits. Significant correlations were also found for T1DM, as well as inflammatory bowel disease, ER+ breast and lung cancer, and asthma [54]. Supporting this finding, in a study with 106 COVID-19 patients, Chen et al. reported that diabetes negatively affects viral clearance, as measured by SARS-CoV-2 qRT-PCR [59].

Angiotensin is a prohormone that is activated into angiotensin II by the angiotensin-converting enzyme (ACE). Angiotensin II acts as an agonist for its receptor, the angiotensin receptor type 1 (AT1). ACE2, on the other hand, promotes the conversion of angiotensin II into angiotensin 1–7 (Fig. 1), which no longer is capable of activating AT1, but instead binds to and activates the angiotensin receptor type 2 (AT2). While AT1 activation is responsible for the major cardiovascular effects attributed to angiotensin II, inducing vasoconstriction, aldosterone synthesis and secretion, and renal tubular sodium reuptake, AT2 activity promotes vasodilation and natriuresis [60]. By controlling the ratio between angiotensin II and angiotensin 1–7, ACE2 is responsible for balancing the opposing roles of AT1 and AT2.

In a mouse model, SARS-CoV infection reduced pulmonary ACE2 expression [61]. The same disturbance was observed in animals that were injected with the viral spike protein alone, indicating that the binding of ACE2 with the spike protein is enough for its downregulation. Interestingly, in this model, acute lung failure could be mitigated by renin-angiotensin antagonists [61].

### Non-respiratory symptoms of COVID-19

Olfactory and gustation impairment can affect around 20% of diabetic patients [62, 63]. Based on patient histories, altered taste and smell sensing are also a clinical feature in COVID-19. In some cases, altered chemosensation may be the only symptom. As such, it appears certain that the chemoreceptor cells for taste and smell are somehow affected by SARS-CoV-2 infection. The chemoreceptor cells for smell, which are modified neuronal cells, are located in the distal (superior) portion of the nasal cavity, encompassing the superior nasal conche and nasal septum, and the distal (nasal) ends of the cells project cilia into the nasal cavity to ‘sense’ and discriminate between airborne chemicals. However, different single-cell RNA-seq studies indicate ACE2 expression in nasal epithelium is found exclusively in non-neuronal cells [64, 65]. This observation suggests that SARS-CoV-2-related anosmia may be caused by infection of support and stem cells in the olfactory epithelium.

Of note, taste buds, which contain the taste receptor cells (TRCs), are not only embedded within lingual epithelium of fungiform, foliate and circumvallate papillae, but they are dispersed across soft palate, nasopharynx, larynx, upper bronchi and proximal esophagus [66]. In murine tissue, ACE2 is expressed in fungiform and circumvallate papillae and throughout the tongue epithelium. And, of immense relevance to SARS-CoV-2, ACE2 is present on the membranes of type I (salt-sensing) and type II (sweet and bitter sensing) TRCs [67]. It is therefore highly likely that SARS-CoV-2 also gains entry to the TRCs in those sites (Fig. 1). There is, as far as we can ascertain from current literature of the topic, no evidence of immune cells residing within taste buds under normal conditions [68, 69] and therefore the capacity for any direct, early action of immune cells in taste buds to clear the virus is low, allowing the virus to replicate unimpeded and potentially shed so as to cause infection lower down the in the pharynx. Direct infection of the TRCs within the enclosed space of the taste bud itself would also likely disturb the signaling machinery of the TRCs by impacting neurotransmitter signals - diminishing synthesis of neurotransmitters, or causing unregulated neurotransmitter release, inability to clear neurotransmitters adequately leading to desensitization of receptors, and disturbance of sensory afferent neurons and their receptors.

Besides serving as viral reservoirs, we hypothesize that TRCs may be involved in serving as the sentinels for an inflammatory response to SARS-CoV-2 and we propose that early loss of taste predicts a brisk IFN response to virus, clearing of virus from the oral and nasal cavities and a faster patient recovery. The innate immune NOD-like receptor pyrin 3(NLRP) inflammasome [70] in TRCs is very likely activated in response to SARS-CoV-2 infection: several viral components from the original SARS-CoV are known to be sensed as danger-associated ?A3B2 show $132#?>molecular patterns (DAMPs) by the NLRP3 inflammasome [71]. The NLRP activates caspase-1, which converts the pro-cytokines IL-1ß and IL-18 into their active forms. Once activated, the cytokines recruit innate immune cells to the site of infection and cause production of IFN-γ. There is direct evidence that inflammation, IFN and taste are linked because of a well-known clinical observation that IFN therapy is reported to cause altered taste and smell [72]. In mice, two IFN-α receptor subunits IFNAR1 and IFNAR2, their downstream JAK kinases JAK1 and TYK2, and the transcription factors STAT1, STAT2, and IRF-9 are all expressed in normal TRCs, and LPS-induced systemic inflammation increases cleaved caspase levels and TRC death within taste buds [73]. Furthermore, with increased death of TRCs there could be increased viral shedding. No loss of taste therefore may be associated with a weak immune response to the virus and the use of IFN-based therapies [29, 30], as described above, may be both preventing virus gaining an ideal replication target and clearing virus from.

## Consideration for treatment specific to people who have both SARS-COV-2 and diabetes

### General principles of treatment of patients with diabetes

There are approximately 30 million people diagnosed with diabetes in the USA and the vast majority of patients who get a COVID-19 infection will be managed at home. As regards in-patients, in one retrospective study of confirmed COVID-19 adults for whom 570 deaths or discharges were recorded, and for which point-of-care blood glucose test results (BGs) are transmitted and stored, the mortality rate was 28.8% in 184 diabetes and/or uncontrolled hyperglycemia patients, as compared with 6.2% of the remaining 386 patients without diabetes or hyperglycemia [74]. So, hyperglycemia, per se, is clearly associated with increased mortality. To underline the necessity of good glucose control, in a retrospective analysis of more than 7300 hospitalized COVID-19 with and without pre-existing T2DM patients, well-controlled blood glucose, that is, maintaining levels within a range of 3.9 to 10.0 mmol/L, was associated with a significant reduction in composite adverse outcomes and death [75].

Attention to nutrition and adequate protein intake is important. Mineral and vitamin intake need to continue. Vitamin C in particular may have a role in COVID-19 prevention, as have been shown for other infections [76]. Hydration should be maintained and treatment with acetaminophen, humidifier use and continuation of CPAP in those with sleep apnea should continue. It seems reasonable that anti-hyperglycemic agents that are known to result in volume depletion, such as sodium glucose transporter-2 (SGLT2) inhibitors, or hypoglycemia, such as chlorpropamide, should be avoided. Care should also be taken with use of glucagon-like receptor-1 (GLP-1R) agonists. Dose reduction or discontinuation altogether may be needed to prevent nausea, abdominal distress and dehydration in patients eating and drinking very little, or in patients with gastrointestinal symptoms directly caused by invasion by the virus of the gut. Dipeptidyl peptidase-4 (DPP4) inhibitors (see discussion below) are weak anti-hyperglycemic agents, are not first line glucose lowering drugs, and probably should be stopped in anyone with any indications of volume depletion or reduced kidney function.

Non-hospitalized patients with T1DM as well as T2DM patients using insulin should measure blood glucose and urinary ketones frequently, especially if fever and increasing hyperglycemia occur - as is true during all infections. Frequent changes in dosage and correctional boluses are par for the course in order to maintain normal glucose levels. Hospitalized patients with severe COVID-19 symptoms and concomitant diabetes need escalating frequency of blood glucose monitoring if infection signs worsen. Subcutaneous insulin via a basal-bolus regimen, where possible, in hospitalized patients, with continuous insulin drip if in an ICU setting are the preferred methods to control blood glucose levels. Oral agents especially metformin and SGLT2 inhibitors should likely be stopped. Biguanides, of which metformin is the only one in use for patients with T2DM and pre-diabetes, are known to increase lactic acid production in the gut, which is then normally cleared in liver. However, as recently described by two independent groups, the human gut epithelium, which contains transmembranal ACE2, is a site of COVID-19 infection that supports viral replication and spread [77, 78], and therefore the expectation would be that endogenous lactic acid levels are already high in gut, as well as other organs, due to infection, which could make the risk of lactic acidosis more likely in the presence of metformin. This might also have implications for lung function: acidosis would impede metabolism and cellular function in infected alveolar cells and T lymphocytes at a time of most vulnerability (see Discussion above).

To reiterate, any drug use that might facilitate dehydration would seem unwise. Increasing thrombosis, especially venous thromboses and micro vascular thromboses in many organs including in pulmonary vasculature [79], and a hypercoagulable state with low platelets [80] are now being reported to occur in extremely sick COVID-19 patients; dehydration would be seriously detrimental under those conditions.

### Specific conundrums in SARS-CoV-2 management in the setting of diabetes

**(a) Chloroquine (CQ) and Hydroxychloroquine (HCQ).** There has been much discussion on the use of CQ and HCQ as therapy for COVID-19 itself, and It is worth remembering that HCQ is an approved treatment, though not first line, for diabetes since 2014 in India. When used for malaria prophylaxis and for treating autoimmune arthritis, such as rheumatoid arthritis and systemic lupus erythematosus (SLE), patients appeared to have reduced risk of T2DM, though in those conditions risk may be reduced for other reasons besides use of the drugs (less obesity, more attention to nutrition, for instance). It was found that HCQ treatment led to less insulin requirements in T2DM, without altering C-peptide levels. This would imply that HCQ reduces clearance of insulin, and not that it increases its secretion [81]. Clinical case reports of its use in T1DM would seem to bear this out because improved glycemic control in the setting of reduced insulin requirements have also been reported in that condition. Additionally, there are reports that CQ improves insulin sensitivity through activation of AKT, which would result in greater glucose uptake and glycogen synthesis [82]. An additional piece of the puzzle relates to adiponectin. It was found that use of the HCQ and CQ leads to increased adiponectin levels in circulation, which is well-described to have favorable effects on glucose uptake/utilization because of its insulin-sensitizing effects. Adiponectin also has anti-inflammatory and anti-apoptotic effects that one could hypothesize would have added benefits during COVID-19, or indeed, any viral infection [83].

As regards its anti-inflammatory properties, HCQ use leads to a significant decrease in the production of pro- inflammatory markers and cytokines, which is why it is a successful disease modifying anti-inflammatory agent in autoimmune diseases. Based on in vitro studies, it changes the pH of acidic intracellular organelles including endosomes/lysosomes, essential for the membrane fusion and it was believed that both the agents could be effective tools against SARS-CoV. And data, again from in vitro work, seemed to indicate that HCQ might be cytotoxic for Sars-CoV-2 [84]. But clinical trials using HCQ have now reported either no benefit or increased mortality due to cardiotoxicity with use of HCQ, with or without azithromycin [85]. Many studies are still on-going, which should give clarity to the use of these agents in SARS-CoV-2. Decreasing certain cytokines, and/or lowering intracellular pH in organelles (which might result in diminished autophagy) might be detrimental when one wants to accelerate clearance of virus.

**(b) Angiotensin converting enzyme inhibitors and angiotensin type 1 receptor blockers (RAAS inhibitors).** Diabetes, hypertension and kidney disease coexist, and many such patients are prescribed RAAS inhibitors because they have well-proven beneficial effects in protecting the kidney and myocardium, and in lowering blood pressure. And while ACE2 expression in lung is increased in diabetes, as stated above, it is not clear that membrane-bound ACE2, which is necessary for SARS-CoV-2 spike protein attachment, is actually increased. There are suggestions in the literature that RAAS inhibitors increase ACE2 but again there is a paucity of data to suggest that these have any effect on lung-specific or nasal-specific expression of transmembranal ACE2. Vaduganathan et al. have written a thorough review on the topic of RAAS inhibitors in patients with SARS-CoV-2 infection [86]. In short, however, the European Society of Cardiology, Council on Hypertension; the American College of Cardiology; the American Heart Association; the Heart Failure Society of America; and the American Society of Hypertension have all released similar statements advising against discontinuing such medications as sudden withdrawal in cardio-renal stable patients who are already under severe stress from infection could lead to clinical instability and decompensation, for no other reason than a response to mere hypothetical concerns.

**(c) DPP 4 inhibitors.** Of great interest in virology, membrane bound human (but not mouse) DPP4 is a functional co-receptor for MERS-CoV [53], though not for SARS-CoV-2. DPP4, also known as CD26, is a type II transmembrane glycoprotein, expressed in many tissues, including the immune cells and non-ciliated bronchial epithelial cells. It also is shed from cell membranes and is therefore present in circulation. In the last 3 decades it has been the subject of much research because of its ability to cleave alanine and proline in the penultimate position of peptides, chemokines and bioactive compounds, resulting in either inactivation or activation of its substrates. DPP4 is well known to diabetes researchers and medical practitioners because it inactivates the well-studied gut-derived incretins, GLP-1 and glucose-dependent insulinotropic polypeptide (GIP) that are released for enteroendocrine cells in response to food in the gut, and GLP-1R agonists, mentioned above, are now routine treatments for T2DM management. Additionally, their use can lead to weight loss. Incretin action through specific receptors on beta cells in islet of Langerhans is responsible for about 50% of the secreted insulin after eating [87, 88]. Levels of active, full length GLP-1 and GIP are increased when DPP activity is eliminated and, as a consequence, DPP inhibitors are now routine, though not first line, oral agents in managing T2DM by improving insulin secretion. DPP4 also plays a role in immune regulation by activating T cells, upregulating CD86 expression and the NF-kB pathway, chemotaxis modulation, cell adhesion, and apoptosis [89]. Its expression is higher in visceral adipose than subcutaneous tissue, and directly correlates with adipocyte inflammation and insulin resistance. Despite it activity on cytokines, plasma levels of 27 plasma cytokines were unchanged after a four-week treatment with sitagliptin, a DPP4 inhibitor [90], and another inhibitor, vildagliptin, did not affect ex vivo cytokine response or function in lymphocytes taken from T2DM patients [91]. Review of the literature does not support any meaningful changes in immune function with DPP inhibition in humans and therefore it is not likely to play a role in treating COVID-19. Additionally, despite DPP4 being a co-receptor for MERS-CoV data are not forthcoming that point to DPP4 inhibitors (saxagliptin, sitagliptin, vildagliptin) sterically interfering with or modifying MERS-CoV binding to subunits of DPP4 [53]. The use of DPP4 by coronavirus seems to be simply due to coronavirus hitching a ride on an abundant target in epithelial cells, unrelated to DPP4’s proteolytic activity.

**(d) HMG-CoA Reductase Inhibitors.** Many T2DM patients are taking statins to control LDL levels and prevent atherosclerotic heart disease. In fact, The American College of Cardiology, the American Heart Association and the American Diabetes Association all have issued recommendations that anyone aged 40 and older with diabetes be prescribed statins: these should be continued during COVID-19 infection. HMG-CoA reductase inhibitors upregulate ACE2 as part of their function in reducing endothelial dysfunction and have many other pleiotropic effects, independent of their lipid-lowering effects. This class of compounds, based on observational studies, may actually reduce mortality in hospitalized patients with influenza and pneumonias, and acute lung injury [92]. There is also anecdotal evidence from Sierra Leone that statins were beneficial during its Ebola outbreak and it has been proposed that these agents have a potential role in managing patients with severe COVID-19 [93].

**(e) Steroids Use.** Steroid use will exacerbate hyperglycemia. But because very high cytokine and CRP levels are routine findings in SARS-CoV-2, steroids have been used in severely ill patients, to suppress the cytokine storm: however, they have not been shown to decrease mortality and theoretically might even slow viral clearance. Even in the setting of adult respiratory distress syndrome with SARS-CoV-2, there is no evidence to suggest they provide any therapeutic benefit. The National Institute of Allergy and Infectious Diseases has issued guidelines on steroid use in certain circumstances during COVID-19 [94]. Use of intranasal steroids, such as fluticasone for allergies, also should be discontinued, until data emerges to say otherwise. It is not yet reported if intranasal steroids enhance viral replication in olfactory epithelium or whether people who use them on a consistent basis are more likely to get infected; there are theoretical reasons why this may be so.

## Conclusions

Individuals with diabetes are at increased risk for infections and, once acquired, generally get more severe infections, and have much greater increase in mortality, compared to non-diabetic patients. This is certainly proving the rule with SARS-CoV-2. It also transpires that 2 coronavirus co-receptors, ACE2 and DPP4, are well-established actors within metabolic and inflammatory pathways, and renal and cardiovascular physiology, and have been front and center in diabetes and metabolic research: ACE2 is a co-receptor for SARS-CoV-2 while DPP4 is a co-receptor for MERS-CoV. The medical and economic consequences of the SARS-CoV-2 epidemic require ongoing and real-time adaptation of protocols and standard medical procedures to deliver diabetes care, and bring SARS-CoV-2 under control so as to lower morbidity and mortality for all, including people who have diabetes and the elderly while the human population waits with breath that is bated for a vaccine.

## Data Availability

Non applicable.

## Abbreviations

ACE, ACE2: Angiotensin-converting enzyme, angiotensin-converting enzyme 2
APN: Aminopeptidase N
AT1, AT2: Angiotensin II receptor type 1, angiotensin II receptor type 2
BG: Blood glucose
COVID-19: Coronavirus disease 2019
CQ: Chloroquine
DAMP: Danger-associated molecular pattern
DPP-4: Dipeptidyl peptidase-4
GIP: Glucose-dependent insulinotropic polypeptide
GLP-1R: Glucagon-like peptide-1 receptor
HCQ: Hydroxychloroquine
IFN: Interferon
IFNAR: Interferon alfa receptor
IL: Interleukin
IRF-3: Interferon regulatory transcription factor
LPS: Lipopolysaccharide
MERS: Middle Eastern respiratory syndrome
NLRP: NOD-like receptor pyrin
RAAS: Renin-angiotensin-aldosterone system
SARS: Severe acute respiratory syndrome
SGLT2: Sodium-glucose co-transporter 2
T1DM, T2DM: Type 1 diabetes mellitus, type 2 diabetes mellitus
Tfh: T follicular helper
Th1, Th17: T helper type 1, T helper type 17
TRC: Taste receptor cell
Treg: T regulatory
